# Hemodynamic Effects of Methamphetamine and General Anesthesia

**DOI:** 10.1155/2022/7542311

**Published:** 2022-02-17

**Authors:** Keyvan M. Safdari, Curtis Converse, Fanglong Dong, Nickolas Alan MacDougall, Kevin Hyer, Alec Runyon, Haley Ahlering, Mark E. Comunale

**Affiliations:** ^1^Arrowhead Regional Medical Center, 400 N. Pepper Ave., Colton, CA 92324, USA; ^2^California University of Science and Medicine, 1501 Violet St., Colton, CA 92324, USA; ^3^Western University of Health Sciences, Graduate College of Biological Sciences, 309 E. Second St., Pomona, CA 91766-1854, USA; ^4^Loma Linda University Medical Center, 11175 Campus St., Loma Linda, CA 92350, USA

## Abstract

**Design:**

A retrospective analysis of all anesthetic records at our institution over a two-year period was performed. *Setting*. Operating room cases under balanced anesthesia. *Patients*. All patients with ASA class I or II, who did not have trauma or were initially admitted to ICU, aged 18–65, without preexisting cardiac, renal, or pulmonary disease. Patients were divided into three groups: those acutely positive for methamphetamine within 48 hours of surgery (*n* = 137), those positive for methamphetamine between 48 hours and 7 days of surgery (*n* = 69), and randomly selected controls who were negative for methamphetamine within 7 days of surgery (*n* = 159). *Measurements*. Intraoperative hemodynamic instability was defined as either a drop of more than 40% in MAP for greater than 5 minutes or requirement for significant amount of vasopressors. *Main Results*. Among the patients who were acutely positive for methamphetamine within 24 hours, 31.4% met the criteria for hemodynamic instability within the first hour of general anesthesia, compared to 26.1% of the subacutely positive patients and 6.3% of controls (*p* < 0.0001). This was despite lower doses of anesthetic medications in the acutely and subacutely positive groups.

**Conclusion:**

Patients who present to the operating room with a positive urine drug screen for amphetamines within 2 days of surgery are at increased risk of hemodynamic instability. Postponing surgery up to 7 days somewhat decreases this risk, but not to the levels of patients who do not use methamphetamines.

## 1. Introduction

The use of illicit methamphetamine is endemic in our area and among our patient population. A survey of our patient data shows an incidence of 5%, compared to the national average of 0.4% [1]. Deleterious effects of acute and chronic methamphetamine abuse have been well documented and include hypertension, aortic dissection, coronary artery spasm, myocardial infarction, cardiomyopathy, and pulmonary hypertension [2–7]. However, there is little in the literature regarding the effect of general anesthesia on patients who are positive for amphetamines upon arrival to the operating room. Four small retrospective case studies on a total of 11 patients taking prescription amphetamines for either ADHD or narcolepsy concluded that continuing prescription doses of these medications preoperatively caused no adverse consequences [8–11]. A recent prospective study that compared 14 pediatric patients with ADHD on prescription amphetamines had no significantly increased risk of intraoperative hypotension or instability during induction when compared to 34 control patients [12]. However, the existing body of evidence has only examined prescription doses of regulated amphetamines, not illicit amphetamines taken in the high doses that abusers are more likely to be using. Our subjective experience has been that a significant number of these patients become hemodynamically unstable under general anesthesia for elective and semielective surgeries, despite being “nontoxic” appearing on the day of surgery (i.e., without tachycardia, hypertension, acute psychosis, or other symptoms of acute intoxication). Our aim in performing this retrospective analysis is to determine whether our impression is correct, and to be able to advise our surgeons and patients regarding possible increased risk of general anesthesia in nontoxic, methamphetamine-positive patients. Our hypothesis is that patients who are acutely positive for methamphetamines are more hemodynamically unstable than controls and are more likely to require vasopressors to maintain a safe blood pressure intraoperatively.

## 2. Materials and Methods

We obtained permission from our institution's IRB, with informed consent waived to perform a retrospective study. Paper anesthesia records were reviewed for a two-year period between 1 January 2014 and 31 December 2015. All patients aged 18 to 65 years who were positive by urine drug screen within 48 hours or between 48 hours and 7 days of surgery undergoing balanced anesthesia met inclusion criteria for the acute intoxication and subacute intoxication groups, respectively. The test used in our laboratory is a urine drug screen, reporting qualitatively positive for any urine concentration greater than 1,000 ng/dL. The test does not distinguish between methamphetamine and other amphetamines. 200 controls were chosen at random and 159 of these were suitable comparisons based on the exclusion criteria. The investigator selecting controls was blinded to the anesthetic record to remove bias. To reduce confounding, patients admitted to the intensive care unit, trauma service, or burn service, patients with history of cardiac disease (including hypertension), end-stage renal disease, chronic obstructive pulmonary disease, diabetes mellitus, patients receiving etomidate or ketamine, surgeries lasting less than one hour, patients receiving total intravenous anesthesia (TIVA), patients positive for cocaine, heroin, or LSD, and any patients categorized as ASA class III or IV were excluded, unless the only reason for ASA class III or IV was methamphetamine abuse. TIVA specifically was excluded since it makes up a very small percentage of the cases performed at our hospital (about 1.9% of the cases screened for inclusion). Hemodynamic instability was defined as at least 40% drop in mean arterial pressure for at least 5 minutes, or requirement of 300 mcg or more of phenylephrine, 15 mg or more of ephedrine, or any amount of epinephrine or vasopressin during the first hour of general anesthesia. Since there is no universally agreed-upon definition of intraoperative hemodynamic instability, our blood pressure criterion was decided upon after reviewing other studies investigating intraoperative complications in which blood pressure was investigated. We chose a cutoff of 40% rather than the 33% determined by Goldman et al. and the 20% used by Carlson et al. because of our clinical impression that patients who are amphetamine-positive tend to have more pronounced hypotension than other patients, and lower than the 50% decrease used by Monk et al. because of concern that this value would be too insensitive [13–15]. We chose to use a proportional cutoff rather than a fixed pressure value to account for baseline abnormalities in blood pressure. All patients received propofol and either sevoflurane or isoflurane, with or without nitrous oxide. The total doses of intravenous anesthetics and narcotics, as well as the maximum and average doses of inhaled anesthetics, were recorded. All anesthetics were administered by the same group of anesthesiologists, CRNAs, and residents in a single county hospital.

All statistical analyses were conducted using the SAS software for Windows version 9.4 (Cary, North Carolina, USA). Descriptive statistics were presented as means and standard deviations for continuous variables, along with frequencies and proportions for categorical variables. Analysis of Variance (ANOVA) tests were conducted to assess the difference of continuous variables among the three groups. Chi-square tests were conducted to assess the association between categorical variables and the three groups. All statistical analyses were two-sided. A *p*-value < 0.05 was considered to be statistically significant.

## 3. Results

**Figure 1:**
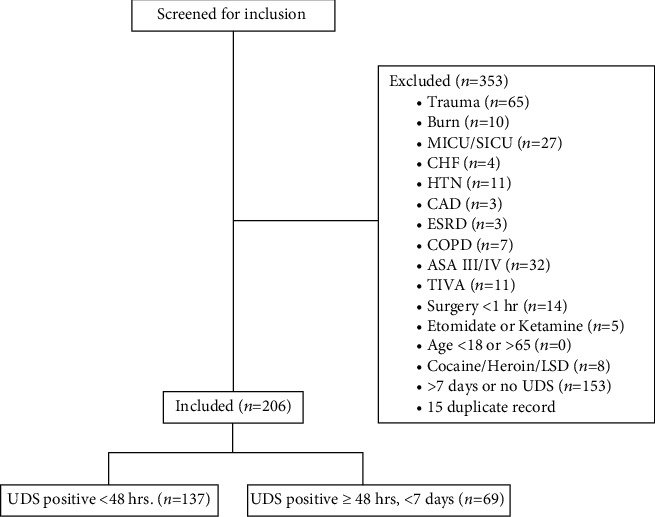
Inclusions and exclusions. CHF: congestive heart failure. COPD: chronic obstructive pulmonary disease. ESRD: end-stage renal disease. HTN: hypertension. LSD: lysergic acid diethylamide. MICU/SICU: medical intensive care unit/surgical intensive care unit. TIVA: total intravenous anesthesia. UDS: urine drug screen.

Out of more than 20,000 cases reviewed, a total of 559 patients were positive for methamphetamine within 7 days of surgery during the study period. 353 met the exclusion criteria. 206 were included in the final analysis, 137 in the acutely intoxicated group, and 69 in the subacutely intoxicated group. See Figure 1 for a diagram of exclusions and inclusions. There were some demographic differences between the three groups, with amphetamine-positive patients tending to be slightly older and having higher ASA class (II vs. I). See Table 1 for a summary of baseline characteristics. Contrary to our expectations, patients who tested positive did not receive higher doses of anesthetics or narcotics and, in fact, patients in the acutely intoxicated group received less propofol for induction than either of the other groups (*p* = 0.001) (Table 2). At the start of surgery, patients in the acute group had higher systolic, diastolic, and mean arterial blood pressure and heart rate than the other two groups. The blood pressure differences disappeared after onset of anesthesia, but heart rate difference persisted. Of the acutely intoxicated patients, 31.4% met our criteria for hemodynamic instability during the first hour of general anesthesia, compared to 26.1% in the subacute group, and 6.3% in the control group (*p* < 0.0001). As there was no prior literature to guide our sample size, we were unable to perform a power analysis prior to the study. Retrospectively, we were able to calculate that the sample size needed to detect the difference we found between the subacute and control groups with 80% power is 49 patients per group, far less than our sample size. In the acute group, 6 of those (14.0%) met the definition due to hypotension, 28 (65.1%) due to vasopressor use, and 9 (20.9%) due to both. This is compared to 0 (0%), 17 (94.4%), and 1 (5.6%) in the subacute group and 2 (20%), 8 (80%), and 0 (0%) in the control group (Table 3). Additionally, among the patients who received phenylephrine or ephedrine, higher doses were required to maintain normal blood pressure among those who were methamphetamine-positive compared to controls (905 ± 761 mcg, 951 ± 718 mcg, and 550 ± 277 for phenylephrine in acute, subacute, and negative, resp., p 0.028; 29.2 ± 28.8, 14.3 ± 10.1, and 8.6 ± 4.1 for ephedrine in acute, subacute, and negative, resp., *p* = 0.016), and only those in the acutely intoxicated group required epinephrine or vasopressin (Table 3). There were no instances of cardiovascular or cerebrovascular events or death within 30 days in any of the study's patients, nor was there report of inadequate anesthesia in the anesthetic record or operative notes.

## 4. Discussion

Our results revealed significantly increased incidence of hemodynamic instability in patients who tested positive for amphetamines by UDS within 48 hours of surgery, compared to patients who tested positive between 48 hours and seven days, and patients who tested negative within a week of surgery. In addition to demonstrating that patients who are acutely toxic from methamphetamine abuse are at significantly increased risk of instability with general anesthesia, our results indicate that patients need more than just a few days of abstinence to return to baseline risk. This could possibly be due to depleted catecholamine reserves taking longer than seven days to replenish, or due to the long-lasting effects of high-dose methamphetamines on the cardiovascular system, specifically cardiomyopathy and pulmonary hypertension [2, 13–16]. In support of this assertion, we note that patients who were outside of the timeline for acute intoxication but with recent history of use were at increased risk compared to nonusers, but not as high as those using amphetamines in the two days prior to surgery.

Patients who abuse methamphetamines are at significant risk of developing several different types of cardiomyopathy including dilated, hypertrophic, and stress-induced cardiomyopathy (Takotsubo and reverse Takotsubo) [17–20]. A study performed by Neeki et al. at the same hospital from which we obtained our data revealed that 38% of the 590 patients aged 18–50 years admitted over a 10-year period with a diagnosis of cardiomyopathy had history of methamphetamine abuse [21]. Another study by Yeo et al. found a similar 40% prevalence of methamphetamine abuse among 107 young patients with a new diagnosis of “idiopathic” cardiomyopathy [22]. There seems to be a relationship between chronic use with more global cardiomyopathy which is less reversible when compared to other causes of cardiomyopathy. Short-term users tend to develop a stress-induced, regional cardiomyopathy that is more prone to recovery after several weeks of abstinence from methamphetamine [23, 24].

Methamphetamine use is also a well-known cause of pulmonary hypertension, which could contribute to hemodynamic changes during general anesthesia. In a 2006 study, patients with a diagnosis of idiopathic or primary pulmonary hypertension were ten times more likely to have a history of methamphetamine abuse than the general population [7]. The association between amphetamine-based diet pills and pulmonary arterial hypertension has also been well studied [25].

Our results contrast with those of other studies of amphetamines and their effect on general anesthesia. These four small case studies (total of 11 cases) and single case control study (14 patients in investigation arm, 34 in control) of patients chronically taking prescription amphetamines for ADHD or narcolepsy did not demonstrate significant hemodynamic instability under general anesthesia [8–12]. The differing results are likely explained by the fact that these patients were taking therapeutic doses of the medication rather than the unpredictable and much higher doses that come with recreational use of illicit amphetamines. A report from the National Highway Traffic Safety Administration states that prescription doses of methamphetamine are typically 2.5–10 mg/day, with a maximum dose of 60 mg/day, while illicit methamphetamine is usually consumed in doses of 100–1000 mg/day, with heavy users consuming as much as 5000 mg/day [26]. Our results are also different from a previous study evaluating stability of patients who tested positive for cocaine on the day of elective surgery [27]. This study found no increase in the incidence of blood pressure or heart rate deviation from baseline, nor in the amount of vasoactive medication or fluid boluses given. The definition of blood pressure deviation for this study was similar to that used in our study (40% deviation in MAP either above or below preoperative baseline) and revealed an incidence of deviation very similar to that seen in our amphetamine-negative group (7.5% in cocaine positive, 10% in cocaine negative, vs. 31.4% in acutely methamphetamine-positive, 26.1% in subacutely methamphetamine-positive, and 6.3% in methamphetamine-negative). The difference between these findings and ours may be accounted for by the short elimination half-life of cocaine compared to methamphetamine (10.1 hours for methamphetamine, 0.8 hours for cocaine) [26]. Another possibility is that cocaine's cardiovascular effects are more apparent in the acute setting, with less chronic manifestations, though there is evidence of ventricular hypertrophy with prolonged use. Yet a third reason may lie in cocaine's direct anesthetic action on the cardiovascular system, which is not blunted by general anesthesia, whereas the sympathomimetic actions are [28].

The importance of our study is in identifying patients who are not yet medically optimized because of acute or subacute methamphetamine intoxication, as general anesthesia may lead to unsafe intraoperative deviations in blood pressure. There is ample evidence in the literature regarding intraoperative hypotension and negative postoperative outcome. Goldman et al. reported four decades ago that a greater than 33% decrease in SBP from baseline for more than 10 minutes results in increased risk of death due to perioperative cardiac events [29]. More recently, it has been shown that there exists a significant association between duration of intraoperative hypotension and postoperative complications, including renal failure, myocardial infarction, and 1-year mortality, among other meaningful endpoints [27, 30, 31]. It is interesting to note that there were no ischemic events (stroke, myocardial infarction, etc.) or death within 30 days in any of the groups, including in those patients who received treatment for hemodynamic instability. This may be due at least in part to the fact that our study was limited to relatively healthy patients (ASA I and II) without preexisting cardiac or renal disease that would typically put them at risk for such complications. It is also possible that prompt reaction to intraoperative hemodynamic instability may have mitigated these effects.

One of the major limitations of our study is its retrospective design. While it would be informative to have a prospective, blinded trial, we believe that it would be unethical to withhold drug screen status from the anesthesiology team, as their level of preparedness is dependent upon anticipating risks specific to their individual patients. However, the lack of blinding to the amphetamine status of the patients may have led to an increase in awareness of potential hypotension by the treating anesthesiologist or CRNA, causing more patients in the amphetamine-positive groups to receive vasopressors when they might otherwise not have been given. In support of this possibility, most patients who were classified as hemodynamically unstable by our definition met the criteria by receiving vasopressors, not because of recorded hypotension. Another explanation for this finding could be that the interval between recordings did not allow for every instance of hypotension to be recorded even though treatment was given. In the future, it would be beneficial to follow patients who are amphetamine-positive for complications during the perioperative period to observe for differences in outcomes postoperatively. Future research should also determine whether our findings are replicated when a broader range of patients are included, such as trauma patients and those who are ASA class III-IV and have other comorbidities. While we assume for the purposes of our study that the urine drug screen for amphetamines is specific for acute or subacute intoxication, there are some medications that are known to cause false positive results. Some of these include promethazine, chlorpromazine, trazodone, ranitidine, bupropion, and nasal decongestants, as well as amphetamines taken pharmaceutically for ADHD, narcolepsy, or weight loss [32]. Promethazine is not given frequently in our hospital and it is unlikely that patients taking the other medications in this list were frequent enough to contribute meaningfully to the data. It is our experience that the majority of patients who test positive admit to taking illicit methamphetamines when questioned, though this was not part of the study.

Our choice to limit our screening for our control group to 200 charts was due in large part to our lack of electronic medical record integration with our anesthesia records. At our institution, the anesthesia record is kept on paper and then scanned into the patients' medical records. A fully electronic system would have allowed for more efficient data extraction and may have permitted better matching of controls with the study groups.

## 5. Conclusion

Our results demonstrate that patients who are acutely intoxicated with methamphetamines within 48 hours of surgery but are otherwise healthy and nontoxic are significantly more likely to have hemodynamic instability and require vasoactive medications during the first hour of general anesthesia than patients who do not use methamphetamines. Patients who are positive between 48 hours and seven days have less risk of hemodynamic instability compared to those who are acutely intoxicated but still are at significantly increased risk relative to the general population.

## Figures and Tables

**Table 1:** Demographic summary.

	No meth (*n* = 159)	Subacute positive (*n* = 69)	Acute positive (*n* = 137)	*p* value
Sex				
Female	45 (28.3%)	12 (17.4%)	24 (17.5%)	0.048
Male	114 (71.7%)	57 (82.6%)	113 (82.5%)	
Age	33.7 ± 11.4	36.1 ± 10.4	38.3 ± 11.5	0.002
Age group				
Age 16 to 30	74 (46.5%)	22 (31.9%)	41 (29.9%)	0.004
Age 31 to 40	43 (27%)	24 (34.8%)	37 (27%)	
Age 41 to 50	27 (17%)	16 (23.2%)	38 (27.7%)	
Age 51 to 60	15 (9.4%)	7 (10.1%)	14 (10.2%)	
Age >61	0 (0%)	0 (0%)	7 (5.1%)	
Modified ASA				
1	27 (17%)	6 (8.7%)	6 (4.4%)	0.002
2	132 (83%)	63 (91.3%)	131 (95.6%)	
Surgery performed				
GI	0 (0%)	1 (1.5%)	1 (0.7%)	Not applicable
GYN	0 (0%)	0 (0%)	1 (0.7%)	
General surgery	26 (17.2%)	10 (14.7%)	14 (10.4%)	
OMFS	16 (10.6%)	7 (10.3%)	3 (2.2%)	
Ophtho	0 (0%)	0 (0%)	1 (0.7%)	
Ortho	107 (70.9%)	50 (73.5%)	113 (83.7%)	
Podiatry	1 (0.7%)	0 (0%)	0 (0%)	
Urology	1 (0.7%)	0 (0%)	2 (1.5%)	
Inhaled agent				
Isoflurane	0 (0%)	1 (1.5%)	0 (0%)	Not applicable
Sevoflurane	159 (100%)	68 (98.6%)	136 (100%)	Not applicable
N_2_O status				
Did not receive N_2_O	86 (54.1%)	38 (55.1%)	84 (61.3%)	0.429
Received N_2_O	73 (45.9%)	31 (44.9%)	53 (38.7%)	
Phenylephrine dose mcg	216 ± 200	527 ± 614	568 ± 653	0.003
Ephedrine dose mg	8.6 ± 4.1	14.3 ± 10.1	29.2 ± 28.8	0.016
Epinephrine dose mcg†	Not applicable	Not applicable	102 ± 139	Not applicable
Vasopressin dose units†	Not applicable	Not applicable	5 ± 1	Not applicable
Average sevoflurane/isoflurane concentration %	2 ± 0.5	1.9 ± 0.8	1.9 ± 0.6	0.175
Average N_2_O concentration % (patients who received N_2_O)	51.3 ± 3.3	50 ± 0	50.9 ± 5.1	0.447
Propofol dose mg/kg	2.5 ± 0.7	2.7 ± 0.9	2.3 ± 0.6	0.001
Fentanyl dose mcg/kg	185 ± 65	182 ± 74	177 ± 63	0.549
Dilaudid dose mg/kg (all patients)	0.0374 ± 0.0056	0.00171 ± 0.105	0.00395 ± 0.0069	0.093
Dilaudid dose mg/kg (patents who received dilaudid)	0.0091 ± 0.0054	0.0422 ± 0.1643	0.0120 ± 0.0070	0.119
Morphine dose mg/kg*∗*	0 ± 0	0 ± 0	0 ± 0	0.135

*∗*Zero patients in control and only two patients in each of the investigation groups receive morphine. †Zero patients in control and subacute groups received vasopressin or epinephrine.

**Table 2:** Comparison of clinical outcomes among the three study groups.

	No meth (*n* = 159)	Subacute positive (*n* = 69)	Acute positive (*n* = 137)	*p* value
Blood pressure at 0 min (SBP/DBP (MAP) mean ± SD)	122.7 ± 12.7/68.7 ± 10.3(75.1 ± 12.6)	120.9 ± 15.7/69.8 ± 11.8(77.1 ± 12.8)	128.2 ± 18.6/74 ± 12.7(83.6 ± 14.9)	0.0014/0.0004(<0.0001)
Heart rate at 0 min (mean ± SD)	85.9 ± 9.4	86 ± 12	91.2 ± 13.4	0.0002
Blood pressure between 5 and 60 min (SBP/DBP (MAP) mean ± SD)	109.9 ± 9.7/62.1 ± 9.8(77.2 ± 8.6)	108.6 ± 11.5/62.6 ± 9.2(77.2 ± 8.9)	108.2 ± 10.6/62.8 ± 8.9(77.2 ± 8.6)	0.3766/0.7972(0.9968)
Mean HR between 5 and 60 min (mean ± SD)	75.1 ± 12	78.3 ± 12.1	83.8 ± 15	<.0001
Hemodynamic instability, *n* (%)	10 (6.3)	18 (26.1)	43 (31.4)	<.0001
Hemodynamic instability by MAP criteria only, n	2	0	6	
Hemodynamic instability by medication criteria only, n	8	17	28	
Hemodynamic instability by both criteria, n	0	1	9	

DBP: diastolic blood pressure. HR: heart rate. MAP: mean arterial blood pressure. SBP: systolic blood pressure. SD: standard deviation.

**Table 3:** Medication usage by the three study groups.

	No meth (*N* = 159)		Subacute UDS (*N* = 69)		Acutely UDS positive (*N* = 137)		
	*N*	Mean ± SD	*N*	Mean ± SD	*N*	Mean ± SD	
Amount of phenylephrine (all patients)	45	215.6 ± 199.9	38	527 ± 613.6	75	567.3 ± 652.5	0.0028
Amount of phenylephrine (patients who received phenylephrine)	8	550 ± 277.7	17	951.5 ± 718.5	39	905.1 ± 761.4	
Amount of ephedrine (all patients)	14	8.6 ± 4.1	11	14.3 ± 10.1	12	29.2 ± 28.8	0.0162
Amount of ephedrine (patients who received ephedrine)	3	See footnote	7	17.9 ± 11.1	9	35.6 ± 30.9	
Amount of epinephrine (all patients)	0	Not applicable	0	Not applicable	2	See footnote	Not applicable
Amount of epinephrine (patients who received epinephrine)	0	Not applicable	0	Not applicable	2	See footnote	
Amount of vasopressin (all patients)	0	Not applicable	0	Not applicable	3	See footnote	Not applicable
Amount of vasopressin (patients who received vasopressin)	0	Not applicable	1	Not applicable	3	See footnote	

*∗*Descriptive statistics were not reported for subgroups with 3 or fewer observations.

## Data Availability

Data are available upon request from the Arrowhead Regional Medical Center Internal Review Board.
